# Implementation support structure for the Dutch Health Promoting School program: a multiple case study

**DOI:** 10.1093/heapro/daaf177

**Published:** 2025-11-05

**Authors:** Gerjanne Vennegoor, Patricia van Assema, Maria W J Jansen, Joyce Dieleman, Gerard R M Molleman

**Affiliations:** Academic Collaborative Center for Public Health Limburg, P.O. Box 33, Heerlen 6400 AA, The Netherlands; Department of Health Promotion, Research Institute of Nutrition and Translational Research in Metabolism (NUTRIM), Maastricht University, P.O. Box 616, Maastricht 6200 MD, The Netherlands; Academic Collaborative Center for Public Health Limburg, P.O. Box 33, Heerlen 6400 AA, The Netherlands; Department of Health Promotion, Research Institute of Nutrition and Translational Research in Metabolism (NUTRIM), Maastricht University, P.O. Box 616, Maastricht 6200 MD, The Netherlands; Academic Collaborative Center for Public Health Limburg, P.O. Box 33, Heerlen 6400 AA, The Netherlands; Department of Health Services Research, Care and Public Health Research Institute (CAPHRI), Maastricht University, P.O. Box 616, Maastricht 6200 MD, The Netherlands; Public Health Service Noord-en Oost-Gelderland, Academic Collaborative Center AGORA, P.O. Box 3, Zutphen 7200 AA, The Netherlands; Department of Primary and Community Care, Radboud Institute for Health Sciences, Radboud University Medical Center, P.O. Box 9101, Nijmegen 6500 HB, The Netherlands; Department of Healthy Living, Public Health Service Gelderland-Zuid, P.O. Box 1120, Nijmegen 6501 BC, The Netherlands

**Keywords:** Health Promoting School, implementation support, context, qualitative, Community of Practice, the Netherlands

## Abstract

Support structures are available to schools worldwide for the implementation of Health Promoting School (HPS) programs. To get more insight in these structures, this multiple case study aimed to map variation in levels of support within eight Public Health Service (PHS) regions in the Netherlands and associations with contextual factors. Designed together with a Community of Practice, the study included two rounds of semistructured group interviews (*N* = 1–4 employees; ±3.5 hours per case) and document analysis. Data were collected on eight indicators of the level of support (e.g. intensity and reach) and 24 contextual factors relating to Healthy School Advisers, PHSs, stakeholder collaboration, and the wider context. Scores were assigned for all indicators and factors per region, and patterns were examined. Results showed large variation in the level of support across cases, mainly in intensity of provided support, integration in the PHS, and reach in terms of percentage of certified HPS schools. Some aspects such as advisers’ context sensitivity scored low in all cases. Key contextual factors were related to the PHS: its policy, internal support, capacity, and (structural) budget. Other important factors related to collaboration with regional stakeholders: coordination, division of responsibilities, and communication structure. Structural budget and strategic stakeholder coordination could be improved in all cases. In conclusion, there is much room for improvement toward sufficient and higher quality HPS implementation support for all schools in the Netherlands. To strengthen support, it is important to establish commitment of the PHS organization, strong coordination between stakeholders, and strong national positioning of the HPS program. These conclusions might also apply to other countries.

Contribution to Health Promotion

- We investigated professional support for school health promotion in eight regions in the Netherlands, using group interviews and existing documents.
- Regional professional support differed greatly in the amount of contact per school, organization structure (e.g. separate department), and percentage of schools working on health promotion.
- Main explanations for the differences concerned organizational factors (e.g. policy and budget) and regional collaboration (e.g. role division).
- In addition, all regions need better alignment of the support at each school, structural funding, and clearer coordination between organizations.
- These findings highlight opportunities to advance professional support for school health promotion in the Netherlands and beyond.

## BACKGROUND

Large-scale implementation of health promotion programs continues to be a major challenge to the achievement of consistent effects in local public health practice (Durlak and DuPre 2008, Van Rinsum *et al*. 2019, Grêaux 2023). Due to the significant efforts required, organizations on their own often do not achieve integrated implementation of all program elements as intended by program developers (Allen *et al*. 2017). Implementation support structures on a national, regional, or local level can help stimulate and improve the quality of the dynamic implementation process (Samdal and Rowling 2013, Vinson *et al*. 2017). This may, for instance, include planning tools to provide structure, grants to cover personnel costs and/or materials, and/or health promotion professionals to provide consultancy (McIsaac *et al*. 2016, Kirchner *et al*. 2017).

Implementation support structures may be helpful for any program, but especially for comprehensive health promotion programs. In order to achieve effects on complex public health problems, such as obesity or smoking behavior, comprehensive programs demand changes at multiple levels of the implementation setting (e.g. individual, organizational, and policy) (Plsek and Greenhalgh 2001, Rutter *et al*. 2017). Implementation of such programs can be characterized by the dynamics of complex adaptive systems (Plsek and Greenhalgh 2001, Rosas 2015, Egan *et al*. 2019). This means that the many people, environments, and other components of organizations involved at operational, tactical, and strategic levels are constantly interacting and adapting to changes. This process can be very unpredictable and nonlinear, resulting in a unique context for each implementation process (e.g. different for each neighborhood or each school; Plsek and Greenhalgh 2001, Rosas 2015, Egan *et al*. 2019). Within these complex adaptive systems, implementation is further challenged by intersectoral collaboration (e.g. between healthcare, sports, and education), both in the program implementation and support structure (Jansen *et al*. 2008, Leenaars *et al*. 2018, Grêaux *et al*. 2021).

Programs based on the Health Promoting School (HPS) framework are one of these types of comprehensive health promotion programs that are implemented in complex adaptive systems (Pucher 2015, Turunen *et al*. 2017, Vilaça *et al*. 2019). The HPS framework entails a whole-school approach including not only health education but also healthy school policies; a healthy physical and social school environment; involvement of students, parents, and the community; and health services (Langford *et al*. 2014, WHO 2021b). Implementation of HPS programs in schools around the world often involves stakeholders from multiple sectors, such as local community sports coaches, addiction prevention organizations, youth health care, and local government (Langford *et al*. 2014, Vilaça *et al*. 2019, WHO 2021a). In some of the countries implementing an HPS program, a support structure for schools is in place. The structures vary in their scope (local, regional, or national), type of organizations and professionals involved (e.g. school nurses or health promotion professionals), type of tools offered to schools, and monitoring and certification (Samdal and Rowling 2013, McIsaac *et al*. 2016, Vilaça *et al*. 2019, WHO 2021a).

**Figure 1.**
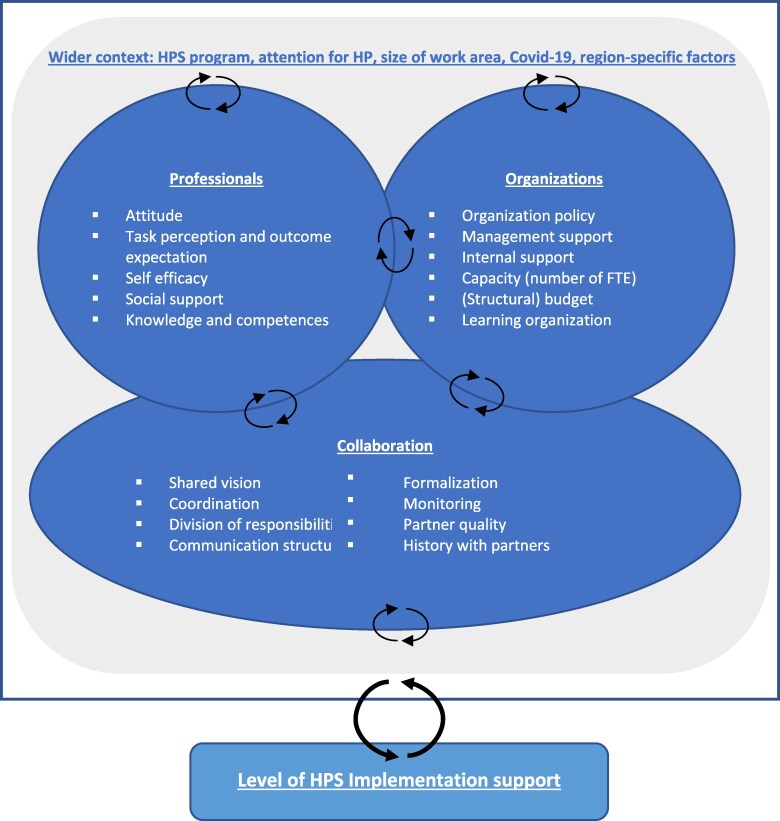
Conceptual model for the evaluation of an HPS implementation support structure. HPS, Health Promoting School; HP, health promotion; FTE, full-time equivalent.

Current types of HPS implementation support structures are not well understood. More research into possibilities to strengthen these structures can help to improve the limited and varied degree of implementation in schools and may reduce current heterogeneity in effects of HPS programs among students (Deschesnes *et al*. 2003, Langford *et al*. 2014, Turunen *et al*. 2017, Gugglberger 2021, WHO 2021a). While clear indicators for the level of implementation support are not available, various existing theories and principles provide guidance on which contextual factors to evaluate. Collectively, as presented in Fig. 1, these suggest 24 factors at four levels: (i) professionals (e.g. attitudes), (ii) organizations (e.g. capacity), (iii) collaboration (e.g. communication structure), and (iv) wider context (e.g. attention for health promotion; Jansen *et al*. 2008, Leurs 2008, Hanleybrown *et al*. 2012, Koelen *et al*. 2012).

This study evaluates the HPS implementation support structure in the Netherlands, where there is a national HPS program since 2010: the Healthy School Program (*Programma Gezonde School*; Jansen and Leurs 2020). At present, implementation support is coordinated nationally (e.g. tools and grants) and executed regionally. The actual support by “Healthy School Advisers” is organized by each of the regional Public Health Services (PHSs) in their own way (Van Koperen *et al*. 2020). Both this autonomy of PHSs and the relatively long history of Dutch HPS implementation support (e.g. Leurs 2008, Boot 2011, Pucher 2015) make it a relevant and interesting structure to evaluate on the current situation and key contextual factors. The present study aims to answer the following two questions: (i) What is the variation in the level of HPS implementation support for schools between eight regions in the Netherlands? and (ii) How and to what extent can differences in the level of support between regions be explained by (interactions between) factors relating to the professionals (i.e. Healthy School Advisers), organizations (i.e. PHSs), collaboration, and wider regional and national context?

## METHODS

### Health Promoting School implementation support structure in the Netherlands

The support structure of the Dutch national HPS program (“Healthy School Program”) consists of a national and regional component. On a national level, the program is funded by the government and coordinated by the National Institute for Public Health and the Environment (*RIVM*), the association of PHSs (*GGD GHOR*), and the education councils for primary, secondary, and secondary vocational education (*PO-Raad*, *VO-raad*, and *MBO Raad*). Together, they offer implementation tools based on core principles of the program, certification, monitoring, coordinator training, and topic-specific and general grants (including a budget as well as a certain number of hours of advice by a regional professional; Van Koperen *et al*. 2020). On a regional level, support is executed by 25 PHSs, which are one of the main stakeholders in (school) health promotion in the region (Neppelenbroek *et al*. 2020). Their work is predominantly funded by local governments, which jointly decide on the amount of and targets for the budget of their PHS. Another source of funding is project based, of which the national program grants are one. The program grants provide a set budget for each school that receives a grant. PHSs employ Healthy School Advisers who support schools in the implementation process, following HPS principles and using the nationally available resources. Apart from Healthy School Advisers, other professionals may also support schools in implementing aspects of whole-school health promotion in collaboration with advisers or independently (e.g. advising on implementation plans or providing health education), depending on the health topic as well as regional and local variation in the preventive infrastructure. These are, for example, youth health care physicians and sexual health professionals employed at PHSs, regional organizations such as addiction prevention, and local professionals such as dieticians or sports coaches.

The content of the Dutch national HPS program was described elsewhere in detail (Van Koperen *et al*. 2020, Vennegoor *et al*. 2023b).

### Study design and setting

This is a multiple case study using mixed methods, but predominantly qualitative methods by means of two interview rounds and document analysis. Written informed consent was obtained prior to participating in the study. Ethical clearance was provided by the Ethics Review Committee of the *Faculty of Health, Medicine, and Life Sciences* of *Maastricht University* (FHML-REC/2020/082.02). The study is part of an extensive evaluation study into conditions for effectiveness of the Dutch national HPS program (Vennegoor *et al*. 2024). For the evaluation study, eight out of 25 regions in the country were selected for participation based on variation in location (in the north, east, south, and west of the country), urbanity (capital region, cities, and rural areas), and willingness to participate. These eight regions were the cases that were analyzed in this multiple case study. A Community of Practice was started in 2020 as a means to facilitate a mutual learning process between research and practice (Wenger 1998, Vennegoor *et al*. 2024). Participants to its semiannual meetings are Healthy School Advisers, team managers, and epidemiologists employed at the eight PHSs, employees of the Dutch national HPS program and of other national organizations in the health and education sector, and scientists. In total, the community has about 75 members, of whom on average 35 participate in each meeting (about half are PHS employees).

### Data collection

#### Selection of indicators and contextual factors

Data collection methods were developed in close collaboration with the Community of Practice. This way, theoretical knowledge among the research team was supplemented by practical knowledge on real-life implementation support for school health promotion and regional contexts. In an iterative process, practice professionals provided information on the type of data (e.g. registration data on implementation at each school) and documents (e.g. regional vision documents) that were generally available at PHSs, and discussions were held on which indicators would capture the implementation support and context as completely as possible.

Based on practice professionals’ input and relevant literature (Leurs 2008, Boot 2011, Hanleybrown *et al*. 2012, Dittrich *et al*. 2014, Neppelenbroek *et al*. 2020, Van Koperen *et al*. 2020, Bartelink *et al*. 2022), eight indicators for the level of HPS implementation support were identified (I1–8; Table 1). Two of these were related to integration of the support: within the PHS organization (e.g. department structure; I1) and within the regional collaborative system (e.g. firmness of coordination role to external partners; I2). Three were related to actual support provided by Healthy School Advisers: the intensity (e.g. time investment per school; I3), quality (e.g. following HPS principles; I4), and context sensitivity (e.g. aligning topic choice to school context; I5). The final three were related to reach of the support: percentage of schools Healthy School Advisers are in contact with (I6), percentage of certified HPS schools (I7), and percentage of schools with at least one national grant for regional support (I8).

**Table 1.** Overview of scores and perceived prominence per indicator and factor (short version).

Indicator/factor		C1	C2	C3	C4	C5	C6	C7	C8
**Level of support**									
*Overall groups*		*Low*		*Middle*			*High*		
I1 Integration in PHS	Score	−−	−	−	−	+	++	++	++
I2 Integration in region	Score	−−	−	+	+	+	++	++	++
I3 Intensity of support	Score	−−	−	+	+	+	++	++	++
I4 Quality of support	Score	−	−	+	++	+	++	++	+
I5 Context sensitivity of support	Score	−	+	++	+	+	−	+	+
I6% of schools in contact	Score	−−	−	+	+	++	++	+	++
I7% of certified schools	Score	−−	+	−	+	−−	−	++	+
I8% of schools with grant	Score	−−	++	++	+	−	+	+	++
**Factors related to the professional (Healthy School Advisers)**									
*Overall*	*Score*	*+*	−	*++*	*++*	*+*	*+*	*+*	*+*
	*Prom.*	*+*	*n.a.*	*+*	*+*	*+*	*+*	*+*	*+*
F1 Attitude	Score	++	+	++	++	+	+	++	+
	Prom.	*n.a.*	*n.a.*	*n.a.*	*n.a.*	*n.a.*	*n.a.*	*n.a.*	*n.a.*
F2 Task perception and outcome expectation	Score	−	−	+	+	−	++	+	+
	Prom.	*n.a.*	*n.a.*	*n.a.*	*n.a.*	*n.a.*	*n.a.*	*n.a.*	*n.a.*
F3 Self-efficacy	Score	*n.a.*	−	*n.a.*	*n.a.*	*n.a.*	*n.a.*	*n.a.*	*n.a.*
	Prom.	*n.a.*	*n.a.*	*n.a.*	*n.a.*	*n.a.*	*n.a.*	*n.a.*	*n.a.*
F4 Social support	Score	++	+	++	++	++	++	*n.a.*	++
	Prom.	*n.a.*	*n.a.*	*n.a.*	*n.a.*	+	*n.a.*	*n.a.*	+
F5 Knowledge and competences	Score	−	−−	+	+	++	−	+	+
	Prom.	*n.a.*	*n.a.*	*n.a.*	*n.a.*	+	*n.a.*	*n.a.*	*n.a.*
**Factors related to the organization (PHSs)**									
*Overall*	*Score*	−	−	−	−	*+*	*+*	*+*	*++*
	*Prom.*	−	−	−	−	*+*	*+/−*	*+*	*+*
F6 Organization policy	Score	−	−	−−	−−	+	+	++	++
	Prom.	−	*n.a.*	*n.a.*	−	−	−	+	*n.a.*
F7 Management support	Score	+	+	−	−	+	++	+	++
	Prom.	−	*n.a.*	*n.a.*	−	−	*n.a.*	*n.a.*	*n.a.*
F8 Internal support	Score	+	−−	+	−−	−	−	++	+
	Prom.	−	−	−	−	*n.a.*	−	*n.a.*	*n.a.*
F9 Capacity (manpower)	Score	−−	−−	−	−	+	+	−	++
	Prom.	−	−	*n.a.*	−	+	−	+	+
F10 (Structural) budget	Score	−−	−−	−	+	++	++	++	++
	Prom.	*n.a.*	*n.a.*	−	−	+	−	*n.a.*	*n.a.*
F11 Learning organization	Score	−	+	++	+	+	++	+	++
	Prom.	*n.a.*	*n.a.*	*n.a.*	*n.a.*	*n.a.*	*n.a.*	*n.a.*	+
**Factors related to the collaboration**									
*Overall*	*Score*	−	−	−	*+*	*+*	*+*	*++*	*++*
	*Prom.*	−	−	−	*+*	*+/−*	*+/−*	*+/−*	*+*
F12 Shared vision	Score	+	−	−−	+	+	−	++	++
	Prom.	*n.a.*	+	+	−	*n.a.*	*n.a.*	−	*n.a.*
F13 Coordination	Score	−	−−	−−	−	+	+	+	++
	Prom.	*n.a.*	−	*n.a.*	*n.a.*	*n.a.*	*n.a.*	*n.a.*	*n.a.*
F14 Division of responsibilities	Score	−	−	−−	−	+	+	+	+
	Prom.	−	*n.a.*	*n.a.*	−	−	*n.a.*	*n.a.*	−
F15 Communication structure	Score	−−	−	+	++	++	+	++	++
	Prom.	*n.a.*	−	*n.a.*	*n.a.*	+	*n.a.*	−	*n.a.*
F16 Formalization	Score	+	*n.a.*	*n.a.*	*n.a.*	++	*n.a.*	*n.a.*	++
	Prom.	*n.a.*	*n.a.*	−	−	−	*n.a.*	*n.a.*	*n.a.*
F17 Monitoring	Score	−−	*n.a.*	+	+	+	*n.a.*	*n.a.*	+
	Prom.	*n.a.*	*n.a.*	*n.a.*	*n.a.*	*n.a.*	*n.a.*	*n.a.*	*n.a.*
F18 Partner quality	Score	−	−	++	++	+	−−	++	++
	Prom.	*n.a.*	*n.a.*	*n.a.*	*n.a.*	*n.a.*	*n.a.*	*n.a.*	*n.a.*
F19 History with partners	Score	++	*n.a.*	*n.a.*	++	*n.a.*	+	*n.a.*	+
	Prom.	*n.a.*	*n.a.*	*n.a.*	+	*n.a.*	*n.a.*	*n.a.*	*n.a.*
**Factors related to the wider context**									
*Overall*	*Score*	*+*	*+*	*+*	*+*	−	*+*	−	*+*
	*Prom.*	*+/−*	*+*	−	*+/−*	*+*	*n.a.*	*+*	*+*
F20 Healthy School Program	Score	*n.a.*	+	++	+	+	+	−	++
	Prom.	*n.a.*	*n.a.*	*n.a.*	+	+	*n.a.*	*n.a.*	+
F21 Size of work area	Score	+	++	++	++	−−	+	−	+
	Prom.	*n.a.*	*n.a.*	*n.a.*	*n.a.*	*n.a.*	*n.a.*	*n.a.*	*n.a.*
F22 Attention for health promotion	Score	+	+	+	+	+	++	+	++
	Prom.	*n.a.*	*n.a.*	*n.a.*	*n.a.*	*n.a.*	*n.a.*	+	*n.a.*
F23 Covid-19	Score	−	−	−	−	−−	−−	−	−−
	Prom.	*n.a.*	*n.a.*	*n.a.*	*n.a.*	*n.a.*	*n.a.*	*n.a.*	*n.a.*
F24 Other region-specific factors	Score	−	−	−	*n.a.*	−	+	+	−
	Prom.	*n.a.*	*n.a.*	*n.a.*	*n.a.*	*n.a.*	*n.a.*	*n.a.*	*n.a.*

An extended version of this table, including descriptions of indicators and factors, is available in Supplementary file S2.

C, case; Prom, prominent factor according to participants; PHS, Public Health Service; n.a., the factor or its perceived prominence was not addressed by participants

To assess regional contexts, a selection of contextual factors was made together with the Community of Practice guided by existing literature (Jansen *et al*. 2008, Leurs 2008, Hanleybrown *et al*. 2012, Koelen *et al*. 2012, Dittrich *et al*. 2014). This was based on combining similar factors, selecting relevant factors, and identifying additional specific factors to (Dutch) HPS implementation support. In total, 24 contextual factors were selected, as presented in Fig. 1. These included five factors relating to the professional (i.e. Healthy School Advisers) (F1–F5; Table 1), six to the organization (i.e. PHSs) (F6–F11), eight to the collaboration (F12–19), and five to the wider context (F20–24).

#### Documents and quantitative data

Prior to the interviews, all possibly relevant documents (e.g. year reports, vision documents, and budget plans) were collected from PHSs’ websites, regardless of their publication year. The interviewer read the documents to obtain preliminary information on the position of HPS implementation support in each region prior to the interview. After interview round two, newly uploaded documents were added. These were complemented by internal documents that were discussed during the interviews, for instance, vacancy forms, roadmaps on way of working, and registration data on the quantity of support.

Quantitative data on the total number of schools in each region in August 2022 were derived from an existing open access database of governmental organization *Dienst Uitvoering Onderwijs* (DUO 2020). The Dutch national HPS program provided data on the number of schools which had ever obtained the program certificate and which had ever received at least one national grant through the program until August 2022.

#### Interviews

Following the eight indicators for the level of support and the 24 contextual factors, interview scripts were prepared. Questions, for instance, included the following: “What department does school health promotion belong to at your PHS?”, “How does a Healthy School Adviser generally first get in touch with a school?”, “Is there support among colleagues for school health promotion?”, “How are responsibilities between partners divided?”, and “Which of the categories […] influences support the most?” (see Supplementary file S1, Parts 1 and 2, for the full scripts).

Two audio-recorded semistructured group interviews were conducted with employees of each of the eight PHSs online via Zoom: one of 2 hours between January and August 2021 and one of 1.5 hours between June and September 2022 (16 interviews in total). Interviews were taken about 1 year apart due to project planning and limiting participant burden, as most also participated in other parts of the extensive evaluation study (Vennegoor *et al*. 2024). These each included two to four participants who together had a good overview of implementation support at operational, tactical, and strategic levels in their region, except for one PHS where only one employee had a dedicated role in HPS. In total, 21 and 18 professionals participated, of whom 15 in both rounds (*N* = 24 participants in total). Initial participants were coordinators of Healthy School Advisers, after which other relevant stakeholders within the PHS organization were contacted following the snowball method (Atkinson and Flint 2001). Out of all participants, 15 had the function of Healthy School Adviser, seven were part of the team or department management, one was a policy adviser, and one was a youth health care physician. Numbers of years of experience with school health promotion varied between three and 30, with 13 participants having over 10 years of experience.

During the interviews, a shared screen function was used to jointly make a simple overview of important stakeholders and provide an overview of the roles of Healthy School Advisers (Supplementary file S1, Part 3). It was also used at the end of the second interview to show a simplified version of the conceptual framework and jointly reflect on all the topics that had been covered in both interviews (Supplementary file S1, Part 4).

Recordings were transcribed verbatim, after which detailed interview summaries were created using original phrasing but removing duplicate sections and restructuring data according to the indicators and factors. Summaries were sent to participants for a written member check and were approved by all.

### Data analysis

Qualitative data including the eight interview summaries (one per case, covering both interview rounds in that region) and all documents were entered in NVivo 12 Pro software and coded deductively based on the indicators and factors in Table 1. Quantitative data on having obtained certificates and grants were entered in SPSS software (V.27.0; Armonk, NY, USA). Consequently, the analytical table in Supplementary file S2 was created (see Table 1 for a short version). In the table, based on all available data, a score was given to each of the eight levels of HPS implementation support indicators (I1–I8) and 24 contextual factors (F1–F24) for each case. A general score of each of the four categories of factors was given as well, by averaging all scores within the category. For all scores, a choice was made between four options: “−−,” “−,” “+,” or “++”; and if the factor was not addressed, this was indicated with “n.a.”. The option “++” reflects the highest score and “−−” the lowest, reflecting the potential range for an indicator or factor given the current context of school health promotion. For qualitative data (e.g. quality of support), the range was determined based on theoretical and practical knowledge from the research team and all practical knowledge gathered from the Community of Practice and other professionals during the research period. For quantitative data (e.g. number of municipalities), cut-off values were based on quartiles of national distributions. An equal distribution of cases across the four scoring options was not sought, so it was possible that all cases received the same score for an indicator or factor.

Initial scores were determined by the researcher who conducted the interviews, scoring one factor or indicator at a time for all regions to ensure consistency. The scores were supported by a brief qualitative description of the data on that indicator or factor. All participants approved the descriptions through a written member check and agreed with the assigned scores without comment. The analytic table was further completed by indicating with “+,” “−,” or “not addressed” participants’ perception of the association between each of the four categories of factors and each of the separate factors, with the level of support in their region (as collected at the end of interview round two). The analytic table was then discussed with the research team.

Next, variation between cases in indicators for the level of support was evaluated, after which cases were divided into three groups (*low*, *middle*, and *high*). Finally, patterns between groups and scores for and among factors were searched for. These were checked with participants’ perceptions of the associations. Patterns were characterized as clearly distinctive (a clear pattern, which largely matches participants’ perceptions), fairly distinctive (a mostly clear pattern and/or deviation from participants’ perceptions), and overall stimulating (positive scores in all cases and perceived as prominent by participants in at least two cases). Results were discussed in the research team and during a meeting of the Community of Practice.

## RESULTS

The analytical table in Supplementary file S2 presents an overview of all results per case including a description of each indicator and factor, and Table 1 provides a short version of the Supplementary file. The results are detailed below.

### Variation in level of Health Promoting School implementation support

Most variation between cases was observed for integration of the support in the PHS organization (I1), intensity of the support by Healthy School Advisers (I3), and percentage of certified HPSs (I7). Least variation was observed for context sensitivity by Healthy School Advisers (I5). The other four indicators (I2, I4, I6, and I8) showed medium variation. Although each case was assigned a unique combination of scores on the eight indicators, it was possible to distinguish three general levels of support: *low* (two cases), *middle* (three cases), and *high* (three cases). Within each group, cases could also be ordered from slightly lower to slightly higher.

Half of the cases, all in the *medium* and *high* groups, received positive or very positive scores (“+” or “++”) for integration in the PHS organization (I1). While all *medium* and *high* cases have a separate team or department for school health promotion support, the *low* cases do not have any team. The duration of involvement varies, with some involved from the early development of HPS in the country (2002) and some since a few years, but this is not consistent with the assigned scores for this indicator. All *medium* and *high* cases received positive scores for integration of the PHS in the regional collaborative system (I2), of which all *high* cases and one *medium* case have a solid position of the PHS as a key partner in school health promotion. The two *low* cases received negative scores due to the hesitant position of the PHS toward partners.

Regarding support by Healthy School Advisers (i.e. the intensity, quality, and context sensitivity; I3–5), solely the *high* cases stay in touch with a school after a counseling period, and the *low* cases provide only short-term counseling (e.g. one meeting—I3). In all cases, Healthy School Advisers apply the principles of the HPS framework, but they experience differences between Healthy School Advisers within their region in the way of support, for instance, in the tools or activities used and whether or not a workgroup in school is demanded (in three cases perceived as large differences—I4). The *low* cases and one *middle* case do not have employees dedicated to certain health topics (“topic experts”). Context sensitivity (I5) received negative scores in one *low* and one *high* case, where the way of working is not adapted enough to each school context. In one *medium* case, the choice of topic, way of working, and what roles they take on were all adapted to each school context by all Healthy School Advisers. In all other cases, advisers often described context sensitivity as “deciding which step is best to take first,” for instance, based on internal support in the school, staffing allocation, and staff turnover. They most frequently take on the role of navigator, which was mostly explained as informing schools about their options.

With regard to the percentage of schools Healthy School Advisers are in contact with (I6), the *medium* cases and one *high* case indicated that advisers were in contact with a considerable proportion of schools, two *high* cases were in contact with all schools, and the *low* case with a limited or very limited proportion. Two *high* cases and one *medium* case actively recruit schools for HPS, yet actions are limited and not persistent to schools that are not interested at first. Finally, the percentages of schools with a certificate in a region (I7) and at least one grant (I8) varied largely (between 11% and 34% and 16% and 32% respectively). This was not consistent with levels of support, except for the lowest case receiving very negative scores for both of these indicators.

### Factors influencing the level of support

#### Public Health Service organization

The PHS organization as a whole (F6–F11) was *clearly distinctive* between the three groups of cases. Cases with *lower* support had more negative scores for factors in this category and those participants perceived the PHS organization as hindering and vice versa (except for one *high* case in which participants temporarily perceived the organization as hindering due to turbulence).

Among the PHS organization-related factors, organization policy (F6), internal support (F8), capacity (F9), and (structural) budget (F10) were *clearly distinctive* between groups. In all *low* and two out of three *middle* support cases, HPS had a weak or very weak position in PHS policy (F6). In half of the cases, there was or had recently been turbulence in the form of reorganizations and/or changes in policy, and participants in two of the *middle* and *high* cases therefore perceived the policy as hindering. Internal support (F8) was limited or very limited in half of the cases and perceived as hindering in all but one of the *low* and *middle* cases as well as one *high* case. Other departments are often not aware of HPS, and collaboration between departments is limited. Capacity (F9) in the eight cases varied between 0.11 and 2.83 fte per 100 schools and was mainly consistent with lower groups and participants’ perception (i.e. lower cases had less capacity and perceived this as hindering). All cases further indicated that they had no capacity for recruitment of schools and Healthy School Adviser training, and all but one (*high*) case indicated that school demand for support cannot be met. Budget (F10) varies largely as well, in both amount and structure. The four cases with the highest support receive considerable funding and mostly from structural sources (mainly local government), while the lowest cases have a limited and fragmented budget. Budget was only perceived as limiting by participants in two *middle* cases and one *high* case.

A *fairly distinctive* pattern was seen for management support (F7) in the eight cases, as this is very positive only in the *high* case and participants of three out of five *low* and *middle* cases perceive it as hindering (mainly tactical level and not strategic).

No *overall stimulating* patterns were found for the factors in this category.

#### Collaboration

Collaboration with partners was, as a whole (F12–F19), *fairly distinctive* between groups. It was perceived by participants as stimulating in just two cases (*middle* and *high*), of which in one case, it was called “the key.” Participants of the *low* cases perceived collaboration most prominently as hindering, emphasizing that professionals work alongside each other rather than together.

Of the various collaboration-related factors, coordination (F13), division of responsibilities (F14), and communication structure (F15) were *clearly distinctive* between the groups. The four lowest cases have negative or very negative scores for coordination (F13), mainly between tactical and strategic levels of the PHS, local government, and education, but also between PHS and other regional partners such as addiction prevention. This is perceived as hindering in just one *low* case. Division of responsibilities (F14) also received negative scores in the four lowest cases, but there are two *middle* and *high* cases that perceive it as hindering as well. It is often unclear who is supposed to support the school for each health topic, as in some regions other professionals (e.g. from physical activity organizations) act as Healthy School Adviser, either agreed with Healthy School Advisers or not. There were negative scores for communication structure (F15) in *low* cases only and perceived as hindering in one *low* and one *high* case. In the *low* cases, there are almost no recurring meetings with external partners.

Shared vision among partners (F12) in the eight cases was *fairly distinctive* between groups, with very positive scores only in *high* cases, but inconsistencies with participants’ perceptions. Formalization (F16) was also *fairly distinctive*, mainly due to hindrance only being perceived in all three *middle* cases, and only higher cases receiving very positive scores.

An *overall stimulating* pattern was found for history with partners (F19), as participants from half of the cases discussed it during the interviews and all very positive.

#### Professionals (Healthy School Advisers)

Healthy School Advisers were, as a whole (F1–F5), *overall stimulating* in all but one (*low*) case and perceived as such by participants as well.

Two *fairly distinctive* patterns were observed. First, knowledge and competences (F5) showed negative or very negative scores for the *low* cases and one *high* case. In these cases, Healthy School Advisers’ professional backgrounds do not always match their function and/or there are too little experienced advisers. Second, task perception and outcome expectation (F2) showed negative scores for the *low* cases and one *middle* case. This is due to considerable differences between Healthy School Advisers regarding the task perception of balance between the adviser’s effort and that of the school.

Social support among Healthy School Advisers (F4) showed an *overall stimulating* pattern, with the majority of cases receiving very positive scores and advisers in two cases perceiving it as prominent.

#### Wider context

The wider context was, as a whole (F20–F24), *overall stimulating* in the majority of cases, and perceived as such in four, of which in one expressed by participants as “a huge influence.”

Two *overall stimulating* patterns were found. First, the Dutch national HPS program (F20) showed positive scores for the majority of cases and participants perceiving it as stimulating in three. This is mainly related to the wide awareness of the program, the grants and tools, but participants also indicate that a stronger position of the program in the country would be more stimulating. Second, attention for health promotion on a national level (F22) was positive in all cases and perceived as stimulating in one.

### Additional findings

Next to single-factor influence, interactions between factors were frequently mentioned. PHS capacity, management support, and policy turbulence had the most prominent role in interactions with many other factors. Internal support in the PHS and shared vision with partners were influenced the most by other factors. National attention for health promotion and policies were explained to interact mainly with collaboration factors (e.g. division of responsibilities).

Several factors did not display any patterns: learning organization, monitoring, partner quality, Healthy School Adviser attitudes, Healthy School Adviser self-efficacy, size of work area, Covid-19, and region-specific factors.

Over the course of the study period (between the two interviews), small changes were observed within the cases. These were limited to slightly lower or higher capacity and a different department structure. Plans for larger changes, such as new PHS policy or an increase in budget, were discussed in both interviews, but not implemented in the study period.

## DISCUSSION

This multiple case study aimed to examine regional variation in levels of HPS implementation support for schools and associations with various contextual factors in eight out of 25 regions within the Netherlands. Results show that levels of support varied considerably between regions (i.e. the cases). Most prominent variation was related to integration in the support organization, intensity of support by professionals, and reach in terms of percentage of certified HPS schools. This confirms that each region organizes HPS implementation support in its own way, but also shows that the regional autonomy in the Netherlands is reflected by considerable differences in all three types of indicators examined (integration, Healthy School Adviser support, and reach). Thus, schools in one region have access to a distinctly higher level of support than schools in another region. Consequently, there is much room for improvement toward sufficient support for all schools.

The achieved level of HPS implementation support showed strongest associations with factors in the supporting organization (i.e. the PHS). In line with previous findings on the importance of these factors, most distinctive between regions were a strong organizational policy (Rowling and Samdal 2011, Neppelenbroek *et al*. 2020), internal support (Pucher *et al*. 2017), capacity (Neppelenbroek *et al*. 2020, Jourdan *et al*. 2021, Bartelink *et al*. 2022), and budget (Deschesnes *et al*. 2003, Rowling and Samdal 2011). This shows that building an effective implementation support structure for schools requires substantial attention and effort, with commitment of the entire organization. In regions where this was absent, employees experienced their work as an “island” within the organization. Due to low capacity and budget, employees in these regions were also unable to build relations with schools, provided lower quality support, had low partner quality, and had poor coordination of collaborations.

Other clear associations with the level of support in a region were found in collaboration-related factors and mainly in coordination, division of responsibilities, and communication structure. This shows the importance of making collective impact—i.e. jointly creating lasting large-scale change—but also the challenges this poses in coordinating efforts by the many professionals working in HPS support and in achieving mutual reinforcement of these efforts (Hanleybrown *et al*. 2012, ORS Impact and Spark Policy Institute 2018, Fransen *et al*. 2022). In regions where this was not (yet) achieved, professionals mostly worked alongside each other and sometimes even competed to support certain schools. The more successful regions illustrated that it is possible to achieve stronger collective impact, but that these kinds of processes require a considerable amount of time and perseverance from all stakeholders and at all levels (operational, tactical, and strategic) (Pucher *et al*. 2015b, Fransen *et al*. 2022).

There were three main aspects of support that could be improved in nearly every region. Firstly, context sensitivity by Healthy School Advisers could be improved in all but one region. Advisers often did not adapt their approach enough to the school context, and their perspectives on the role of navigator were limited. Previously, context sensitivity has been emphasized as central in the role of professionals supporting schools (Rowling and Samdal 2011, Jourdan *et al*. 2021, Bartelink *et al*. 2022). Future research may examine how to increase Healthy School Advisers’ context sensitivity in order to truly align HPS implementation support with each school context. Secondly, in all regions, there were differences between Healthy School Advisers in their way of supporting schools. These differences seem to be rooted in the wide variety of (educational) backgrounds of professionals, in a variety of task perceptions and competences, and in learning how to support mainly on the job. It would be useful to work toward consensus on the key principles in supporting HPS implementation at a national level. Additionally, recurring reflective sessions among professionals on the application of the principles in practice can be facilitated at a regional level. Thirdly, quantitative indicators for the level of support (e.g. percentage of schools with certificates), as were used in an earlier study (Pucher *et al*. 2015b), did not match the actual level of support in most regions. Previously, such statistics were already shown to not reflect the actual implementation status, since many noncertified schools still implement HPS (Vennegoor *et al*. 2023a). Moreover, regions with lower levels of support often focus more on quantity of support to, for instance, convince municipalities of school interest, while high-level regions are less concerned with this due to the already solid positioning. These results call for more focus on quality of implementation support, both in research and practice.

Two contextual factors can be improved in all regions. Regarding capacity, it was indicated in all regions that school demand could not be met and there was no capacity to actively recruit schools, as was also mentioned in previous literature (Vilaça *et al*. 2019, Neppelenbroek *et al*. 2020, Bartelink *et al*. 2022). This can largely be explained by the fragmented and temporary funding of (support for) HPS programs, leaving insufficient financial room or security (Vilaça *et al*. 2019, GGD GHOR Nederland 2022). Therefore, there is a need for an increased and structural national budget for HPS implementation support, in order to reach all schools. Furthermore, there is room for improvement of coordination at tactical and strategic levels in all regions, focused on clarity on the division of responsibilities. This includes coordination with the education sector, local governments, and other health promotion organizations (Deschesnes *et al*. 2010, Grêaux *et al*. 2021, Jourdan *et al*. 2021, Fransen *et al*. 2022). At a regional level, PHSs can take clearer responsibility in supporting school health promotion by taking on a stronger role toward stakeholders in the region. At a national level, this can be supported by reducing the noncommittal nature of HPS implementation support for regional stakeholders, especially for PHSs and local governments.

Both the social support among professionals and the history with stakeholders stimulated the level of support in all regions. These connections are especially valuable in the fragmented and complex field of school health promotion and deserve continued attention. Additionally, several factors showed no (clear) association with the level of support, but this does not necessarily mean these are not relevant. It is possible that other factors were more prominent or that certain factors are considered “normal,” such as a positive attitude among professionals. One of these factors without association, turbulence, still deserves consideration. This is because turbulence was shown to occur frequently across all levels of support and to have a major influence on many other contextual factors. Given the widespread influence, the impact of turbulence should be carefully considered (Pucher *et al*. 2015b).

### Methodological considerations

The close collaboration between research and practice in the study has ensured suitable data collection to answer the research questions. All participants have approved the interview summary and descriptions in the analytical table for their region. Community of Practice members also emphasized the realistic and recognizable results of the overall study for HPS implementation support in the Netherlands. This confirms the validity of the method and the value of the study design as inspiration for future similar research in other countries. Even though a limited number of PHS employees participated per region, it is expected that the joint comprehensive view of participants at operational, tactical, and strategic levels resulted in an adequate description of the level of support and contextual factors in each region. Perspectives of other regional stakeholders in HPS implementation support were not included since PHS employees are considered the main professionals to support whole-school health promotion (Leurs 2008, Neppelenbroek *et al*. 2020). The choice of group rather than individual interviews resulted in richer data by participants encouraging each other to deepen the discussion. Associated risks (e.g. social desirability) are expected to be limited due to the close-knit and small PHS teams and no indication of influence from hierarchical structures during the interviews. Furthermore, although it was preferred to collect the data in one round of interviews, two rounds had to be conducted due to limits to participant burden. The interviews were eventually conducted 1 year apart because of project planning, but limited changes over time provided additional confirmation of the key observed patterns.

The findings of this study are expected to be generalizable to all 25 regions in the Netherlands because of the variation among the eight regions in location, size of work area, way of supporting schools, and (length of) history of supporting schools and confirmation of generalizability by Community of Practice members. Generalizability to other countries is limited by the large differences in support structures, for instance, regarding the scope (local, regional, or national) and type of professionals involved (Samdal and Rowling 2013, McIsaac *et al*. 2016, Vilaça *et al*. 2019, WHO 2021a). Nonetheless, despite differences between countries, the findings may prompt further discussions at a national, regional, or HPS program level on, e.g. role division in HPS implementation support and (dis)advantages of a structure with regional autonomy. In addition, it is possible that the general patterns in our findings also apply to other countries (Cohen *et al*. 2018), especially patterns that were emphasized in other studies, including the limited level of context-sensitive support and the importance of making collective impact and providing structural funding.

## CONCLUSION

There is much room for improvement toward sufficient HPS implementation support for all schools in the Netherlands, especially in terms of intensity of provided support and integration in the support organization. Additionally, in all regions, improvements can be made in context sensitivity of professionals, comparability of the way of support, and focus on the quality of the support in evaluations. To strengthen the level of HPS implementation support, it is important to establish (i) commitment of the entire support organization, in terms of clear policy, internal support, and structural resources; (ii) strong coordination between stakeholders at strategic, tactical, and operational levels, in order to provide clarity on division of responsibilities; and (iii) strong national positioning of the HPS program to facilitate stakeholder collaboration. Due to the uniqueness of HPS implementation support structures around the world, these conclusions are mainly generalizable to the Netherlands, but might also apply to other countries.

## Supplementary Material

daaf177_Supplementary_Data

## Data Availability

The data underlying this article cannot be shared publicly due to privacy considerations for the study participants, in accordance with ethical approval.
