# Supplementary material for: Implementation support structure for the Dutch Health Promoting School program: a multiple case study
Source: Health Promot Int. 2025 Nov 5;40(6):daaf177. doi: 10.1093/heapro/daaf177 (PMC12596121; doi:10.1093/heapro/daaf177)

**Supplementary file 1 – Interview scripts**

Part 1: interview script round 1

- *Explain aim and type of interview*
- *Explain audio-recording and anonymity*
- *Sign informed consent*
- *Start audio recording*

1. Do you agree to record this conversation?

**Background**

1. What is your position?
2. What is your experience with school health promotion?

**Organization of support**

Public Health Service and Healthy School Advisers

*I would first like to discuss the overall organization of school health promotion within Public Health Service XX, such as the departmental structure, funding and number of employees.*

1. In what year did you start working according to the Healthy School Program? (Integration)
2. What department does school health promotion belong to at your Public Health Service? (Integration)
3. Which positions are part of school health promotion at your Public Health Service? (Capacity)

*Healthy School Advisers, youth health care physicians, etc.*

- 1. Approximately how many people in total?
  2. Approximately how many fte in total?

1. How many Healthy School Advisers are employed? (Capacity)
   1. Approximately how many fte in total?
   2. Do advisers also have other tasks than school health promotion support?
2. How is the work devided over Healthy School Advisers? (Quality of support)

*Geographically, thematically, etc.*

1. What is the background of advisers? (Knowledge and competencies / Learning organization)
   1. Do all advisers participate in the starter training of the Healthy School Program?
   2. Do advisers regularly attend further training? If so, where?
   3. Does some form of intervision take place among advisers?
2. I would like to identify the main funding sources that you use for implementation support. (Budget)
   1. Is there an allocated budget for school health promotion by the municipalities?
   2. What other sources of funding do you use?

*HPS grants, other municipal grants, etc.*

1. Does Covid influence the organizational structure or funding? (Covid)
2. Have there been any major changes in the organizational structure or funding, for example compared to 5 years ago? (Integration)

**Internal and external partners** (Integration region / Partner quality)

*In the following section, I would like to get an overview of the internal and external partners around school health promotion in your region. The focus here is on the partners of Healthy School Advisers. To do this, I would like to ask you to make a simple overview together. We can do that using the whiteboard. [Explanation whiteboard]*

1. What departments do Healthy School Advisers collaborate with within the Public Health Service?

*Health promotion, policy advisers, youth health care physicians, etc.*

1. What other external local and regional partners do Healthy School Advisers collaborate with?

*Community sports coaches, addiction prevention, nutrition specialists etc.*

- 1. In what way?

1. What national partners do Healthy School Advisers collaborate with?

*National Healthy School Program, nutrition centre, etc.*

- 1. In what way?

1. What are the most prominent partners in the overview? Would you like to underline those?
   1. Why are these the most prominent?
2. Looking at this overview, is it complete? If not, which partners are missing?
   1. What do you notice?
3. Is there influence of Covid on this network? Are connections equally strong, and are the same partners involved? (Covid)
4. Have there been any major changes in the network, for example compared to 5 years ago? (Integration region / History with partners)

**Closing**

1. May I invite you again next year for a follow-up interview, to discuss the topics we haven't gotten to now?
2. What did you think of the interview?

Part 2: interview script round 2

- *Explain aim and previous interview round*
- *Explain type of interview*
- *Explain audio-recording and anonymity*
- *Sign informed consent*
- *Start audio recording*

1. Do you agree to record this conversation?

**Feedback first interview**

1. What did you think of the interview summary?
2. Is there anything that is incorrect?

**Type of support by Healthy School Advisers**

*In the previous interview, we didn't talk about what support in schools by advisers looks like. I would like to get a better picture of that.*

1. How does a Healthy School Adviser generally first get in touch with a school? (Percentage of schools advisers are in contact with / Intensity support)
2. How is support then started? (Quality of support)
   1. Do you have a basic process that every adviser follows roughly? Do they adhere to it?
   2. What nuances are made by advisers? Why?

E.g. ask about mapping starting situation, demanding a workgroup, choice of topic.

1. As a team, do you take a broad approach to a school in the support? How? (Context-sensitivity)

(adapting to school population and their needs, the school organization, and the environment to provide adequate support)

1. *Research by a colleague of mine shows that an adviser can take on multiple roles. [Screen on page 5 is shared]* (Context-sensitivity)
2. To what extent do advisers generally take on each of these roles?
3. Which is/are taken on most often by advisers in your Public Health Service? Why?

**Factors relating to individuals**

*I would still like to learn more about the competencies of consultants and the atmosphere among them.*

1. What is the atmosphere among advisers like?
2. Are they positive or negative about implementation support? Why? (Attitude)
3. Do advisers think that the support has the desired impact? (Outcome expectation)
4. Do they themselves derive satisfaction from their work? (Personal (dis)advantage)
5. Do advisers experience adequate support from colleagues in their work? (Social support)
6. Do advisers have sufficient knowledge and competences to adequately support schools? (Knowledge and competences)
7. If not, which are missing? How can they be improved?
8. Do they also feel sufficiently competent? (Self-efficacy)
9. Does this apply to all schools? For what type of schools does it not?

**Additional questions if needed:**

1. ..
2. ..

**Joint reflection**

*[Screen on page 6 is shared]*

1. Which category of factors has the most influence: collaboration between partners, the Public Health Service organization, the Healthy School Advisers, or the broader context? Why?
2. And which of the separate factors you see on the screen do you think has the most influence on the level of support in your Public Health Service region? Why?

**Closing**

1. Are there any topics we didn’t discuss in both interviews that are important to mention?
2. What did you think about the interviews?

*Potential additional questions (determine before the interview which are needed)*

***Factors related to collaboration***

1. *To what extent do you think history with partners plays a role in how support is currently organized in your Public Health Service region? (History with partners)*
2. *To what extent is coordination between the strategic levels of education, municipalities, and Public Health Service in place? (Communication structure / Coordination)*
3. *In what ways are partners collaborating in your region?*
4. *Are there joint meetings? (Communication structure / Coordination)*
5. *Which communication channels are used? (Communication structure)*
6. *Is there a shared vision? If so, is this described in any document? (Shared vision / Formalization)*
7. *Are joint plans made for how to support schools in the region? (Shared vision / Coordination)*
8. *How are responsibilities divided? (Division of responsibilities)*
9. *Do evaluations take place? (Evaluation)*
10. *Is there influence of Covid on the collaboration? (Covid)*
11. *Have there been any major changes in the collaboration, for example compared to 5 years ago? (Integration region)*

***Factors related to the*** *Public Health Service* ***organization***

1. *As an organization, how important does the Public Health Service consider whole-school health promotion? (Public Health Service policy / Leadership)*
2. *What role do you as Public Health Service play in the collaboration around school health promotion in your region? Are you satisfied with this? Why? (Integration region)*
3. *Do you experience support from management for working on school health promotion support? How is that expressed? (Leadership)*
4. *Is there support among PHS colleagues for offering school health promotion support? (Internal support)*
5. *Is there influence of Covid on the Public Health Service organization? (Covid)*
6. *Have there been any major changes in the Public Health Service organization, for example compared to 5 years ago? (Integration region)*

***Factors related to the broader context***

1. *Are there important laws and regulations, within the organization, or locally/regionally/nationally that affect or have affected what you can or cannot do, or in what ways? (Laws and regulations)*

Part 3: Overview of the roles of Healthy School Advisers (used for question 7 in round 2)


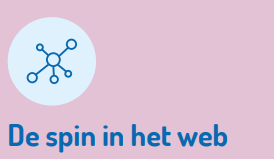

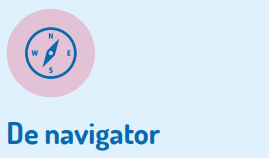


who has expertise and up-to-date knowledge on approaches around health and well-being, and local opportunities and possibilities

who helps make choices that fit the needs, desires and capabilities of the school


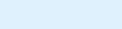


**Navigator**

who connects the school with external partners, such as municipalities or welfare organizations


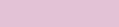


**Linking pin**


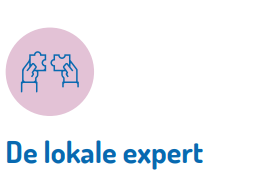


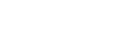

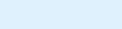

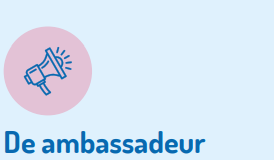

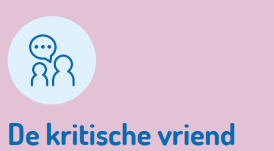


**Local expert**

who enthuses the school to work on health and well-being

who thinks critically in shaping the approach


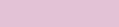


**Critical friend**

**Ambassador**

Part 4: Simplified version of the conceptual framework (used for questions 12 and 13 in round 2)

***
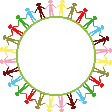
***
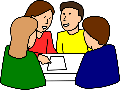


**3. Collaboration with partners**

- Leadership/coordination
- Shared vision
- Division of responsibilities
- Communication structure/planning
- Evaluation/monitoring
- Formalization
- Quality of partners
- History with partners

**1. Healthy School Advisers**

- Knowledge, competences, and trust
- Opinion/attitude
- Support by colleagues
- Expectations about effects/satisfaction among schools/students


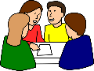

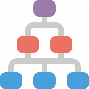


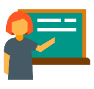

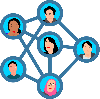


***
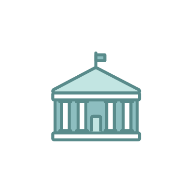
***

**4. Socio-political environment**

- Municipal policy/attention (whole school) health promotion
- Regional policy/attention (whole school) health promotion
- National policy/attention (whole school) health promotion
- Number of inhabitants/municipalities/schools

**2. PHS-organization**

- Public Health Service-policy
- Support from (team) management
- Support from other departments
- Available hours/fte
- Budget
- Learning organization


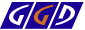


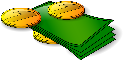

Supplement: daaf177_Supplementary_Data [file daaf177_supplementary_data.zip › Supplementary file 1 - Interview scripts.docx]
