# Supplementary material for: Implementation support structure for the Dutch Health Promoting School program: a multiple case study
Source: Health Promot Int. 2025 Nov 5;40(6):daaf177. doi: 10.1093/heapro/daaf177 (PMC12596121; doi:10.1093/heapro/daaf177)
Supplement: daaf177_Supplementary_Data [file daaf177_supplementary_data.zip › Supplementary file 2 - Analytical table.docx]

**Supplementary file 2 – Analytical table**

| **Indicator**  **/Factor** | **Type of information** | **Source** |  | **Case 1** | **Case 2** | **Case 3** | **Case 4** | **Case 5** | **Case 6** | **Case 7** | **Case 8** |
| --- | --- | --- | --- | --- | --- | --- | --- | --- | --- | --- | --- |
| Level of support | | | | | | | | | | | |
| ***Overall groups*** | |  |  | **LOW** | **LOW** | **MIDDLE** | **MIDDLE** | **MIDDLE** | **HIGH** | **HIGH** | **HIGH** |
|  |  |  |  |  |  |  |  |  |  |  |  |
| **I1 Integration in PHS** | Adopter category Roger's, separate team/ department, general comments on integration in PHS | I, D | *Score* | -- | - | - | - | + | ++ | ++ | ++ |
|  |  |  | *Descr* | Late majority. No separate team or department. Almost no integration in PHS. | Early majority. No separate team or department. Support is limitedly integrated. | Late majority. There is a separate Healthy School team. Support is limitedly integrated. | Early adopter. There is a separate Healthy School team. Support is limitedly integrated. | Early majority. There is a separate Healthy School team. Support is reasonably integrated. | Late majority. There is a separate Healthy School department. Support is fully integrated in the PHS. | Innovator. There is a separate Healthy School team. Support is fully integrated in the PHS. | Early majority. There is a separate Healthy School team. Support is fully integrated in the PHS. |
| **I2 Integration in region** | Firmness of role PHS towards stakeholders, clarity of role PHS towards stakeholders | I, D | *Score* | -- | - | + | + | + | ++ | ++ | ++ |
|  |  |  | *Descr* | The PHS collaborates with stakeholders, but is hesitant. The role of the PHS is very limited. | The PHS collaborates with stakeholders, but is reasonably hesitant. The role of the PHS is limited. | The PHS is an important stakeholder in the region. The role is somewhat limited, but this is developing. | The PHS collaborates with stakeholders, but is somewhat hesitant. The role of the PHS is clear yet not firm. | The PHS is an equal stakeholder in the region. The role of the PHS is clear and reasonably firm. | The PHS is an equal stakeholder in the region. The role of the PHS is clear and firm. | The PHS is an equal stakeholder in the region. The role of the PHS is clear and firm. | The PHS is an equal stakeholder in the region. The role of the PHS is clear and firm. |
| **I3 Intensity of support** | Time investment per school, maintained contact | I, D | *Score* | -- | - | + | + | + | ++ | ++ | ++ |
|  |  |  | *Descr* | Low intensity, limited and short-term support | Low intensity, contact with schools is temporary | Medium intensity, contact with schools is temporary | Medium intensity, contact with schools is temporary | Medium intensity, contact with schools is temporary | High intensity, contact with schools doesn't end | High intensity, contact with schools doesn't end | High intensity, contact with schools doesn't end |
| **I4 Quality of support** | Appointed/no topic experts, following HPS principles, differences among HSAs in way of support | I, D | *Score* | - | - | + | ++ | + | ++ | ++ | + |
|  |  |  | *Descr* | No topic experts. HPS principles are followed. Differences in way of support among HSAs. | No topic experts. HPS principles are followed. Large differences in way of support among HSAs. | No topic experts. HPS principles are followed. Few differences in way of support among HSAs. | Topic experts appointed. HPS principles are followed. Few differences in way of support among HSAs. | Topic experts appointed. HPS principles are followed. Large differences in way of support among HSAs. | Topic experts appointed. HPS principles are followed. Few differences in way of support among HSAs. | Topic experts appointed. HPS principles are followed. Few differences in way of support among HSAs. | Topic experts appointed. HPS principles are followed. Large differences in way of support among HSAs. |
| **I5 Context-sensitivity of support** | Adapting topic choice to context, adapting way of support to context, adapting HSA role to context | I | *Score* | - | + | ++ | + | + | - | + | + |
|  |  |  | *Descr* | Schools independently choose the topic. Way of support is reasonably strict. HSAs mainly take on the role of navigator, seen as informing about options. | Discussion of choice of ropic. Way of support is reasonably adapted to the context. HSAs mainly take on the role of navigator. | Discussion of choice of topic. Way of support is reasonably adapted to the context. HSAs adapt the role they take on to the school. | Schools independently choose topic. Way of support is reasonably adapted to the context. HSAs mainly take on roles of ambassador, navigator, and linking pin. | Choice of topic discussed at part of the schools. Way of support is reasonably adapted to the context. HSAs mainly take on roles of navigator, ambassador, and critical friend. | Schools independently choose topic. Way of support is reasonably strict. HSAs mainly take on roles of navigator, seen as informing about options. | Discussion of choice of topic. Way of support is reasonably adapted to the context. HSAs mainly take on roles of navigator and linking pin, seen as informing about options. | Schools independently choose the topic. Way of support is reasonably adapted to the context. HSAs mainly take on the role of navigator, but also other roles. |
| **I6 Percentage of schools in contact** | Number of schools in contact with, intensity of recruitment for grant application, intensity of recruitment for HPS | I | *Score* | -- | - | + | + | ++ | ++ | + | ++ |
|  |  |  | *Descr* | In contact with a very limited number of achools. Very limited recruitment for grant application. | In contact with a limited number of schools. Limited recruitment for grant application. | Regular contact with a large part of schools. Reasonably active recruitment for grant application. | Regular contact with a large part of schools. Reasonably active recruitment for grant application. | Regular contact with a large part of schools. Active recruitment for HPS and grant application. | Regular contact with almost all schools. Active recruitment for HPS and grant application. | Regular contact with a large part of schools. Reasonably active recruitment for HPS and grant application. | Regular contact with almost all schools. Active recruitment for HPS and grant application. |
| **I7 Percentage of certified schools** | At least one certificate ever: -- 10-15%; - 15-20%; + 20-30%; ++ 30-40% | E | *Score* | -- | + | - | + | -- | - | ++ | + |
|  |  |  | *Descr* | 12% | 21% | 18% | 27% | 12% | 17% | 34% | 27% |
| **I8 Percentage of schools with grant** | At least one grant ever: -- 15-20%; - 20-25%; + 25-30%; ++ 30-35% | E | *Score* | -- | ++ | ++ | + | - | + | + | ++ |
|  |  |  | *Descr* | 16% | 31% | 30% | 29% | 25% | 28% | 30% | 32% |
| Factors relating to the professional (HSAs) | | | | | | | | | | | |
| ***Overall score*** |  |  | *Score* | *+* | *-* | *++* | *++* | *+* | *+* | *+* | *+* |
|  |  | I | *Prom* | + | *n.a.* | + | + | + | + | + | + |
|  |  |  |  |  |  |  |  |  |  |  |  |
| **F1 Attitude** | Attitude towards HPS support | I | *Score* | ++ | + | ++ | ++ | + | + | ++ | + |
|  |  |  | *Descr* | HSAs are very positive about HPS support and actively lobby within and outside of the PHS | Positive attitudes towards HPS support. Difficult for some HSAs to stay positive when school interest is limited. | Very driven and positive team that is very proud of their work and results. | Very positive attitude in the whole team for health and well-being and for HPS support. | Positive attitudes towards HPS support. Difficult for some HSAs to stay positive when school interest is limited. | Positive attitudes towards HPS support. Difficult for some HSAs to stay positive when school interest is limited. | Very driven and positive team that is very proud of their work and results. | Positive attitudes towards HPS support. Difficult for some HSAs to stay positive when school interest is limited. |
|  |  | I | *Prom* | *n.a.* | *n.a.* | *n.a.* | *n.a.* | *n.a.* | *n.a.* | *n.a.* | *n.a.* |
| **F2 Task perception and outcome expectation** | Perception on way of support and differences among HSAs, outcome expectations and differences among HSAs | I | *Score* | - | - | + | + | - | ++ | + | + |
|  |  |  | *Descr* | Differences in task perception on indepencence of schools. | Part of the advisers, who are primarily youth health care physicians, do not perceive necessity of HPS support tasks apart from their regular work. | Whole team perceives necessity to make a difference with their work. Differences in task perception on indepencence of schools. | Whole team perceives necessity of their work. Task perception is clear and unanimous. However, expectations are sometimes different than results. | Differences in task perceptions on way of support that best fits the HSA and the school. | Task perception is clear and unanimous. | Overall task perception is clear and unanimous. Differences in task perception on independence of schools and specific way of support. | Overall task perception is clear and unanimous. Differences in task perception on independence of schools and specific way of support. |
|  |  | I | *Prom* | *n.a.* | *n.a.* | *n.a.* | *n.a.* | *n.a.* | *n.a.* | *n.a.* | *n.a.* |
| **F3 Self-efficacy** | Self-efficacy and differences among HSAs | I | *Score* |  | - |  |  |  |  |  |  |
|  |  |  | *Descr* | *n.a.* | Part of the advisers don't think they are not able to do take on ambassador roles. | *n.a.* | *n.a.* | *n.a.* | *n.a.* | *n.a.* | *n.a.* |
|  |  | I | *Prom* | *n.a.* | *n.a.* | *n.a.* | *n.a.* | *n.a.* | *n.a.* | *n.a.* | *n.a.* |
| **F4 Social support** | Intensity of social support and general atmosphere | I | *Score* | ++ | + | ++ | ++ | ++ | ++ |  | ++ |
|  |  |  | *Descr* | HSAs intensively support and help each other. | HSAs support each other. Bonding of the team is still developing. | HSAs intensively support each other and there is a good atmosphere. HSAs closely collaborate. | HSAs intensively support and help each other. | HSAs intensively support and help each other. | HSAs intensively support each other and there is a good atmosphere. HSAs closely collaborate. | *n.a.* | HSAs intensively support each other and there is a good and open atmosphere. |
|  |  | I | *Prom* | *n.a.* | *n.a.* | *n.a.* | *n.a.* | + | *n.a.* | *n.a.* | + |
| **F5 Knowledge and competences** | Approach to HSA recruitment, relevance of HSA backgrounds, current knowledge and competences | I | *Score* | - | -- | + | + | ++ | - | + | + |
|  |  |  | *Descr* | HSA recruitment limited regarding education and competences. Relevent HSA backgrounds. Somewhat limited knowledge and competences due to limited experience. | Broad HSA recruitment. Relevant HSA backgrounds. Knowledge and competences limited for a part of the advisers due to type of education and limited experience. | Broad HSA recruitment. Various HSA backgrounds, of which most are relevant. Varying knowledge and competences based on experience. | Broad HSA recruitment. Various HSA backgrounds, of which most are relevant. Varying knowledge and competences based on experience. | Broad HSA recruitment. Various HSA backgrounds, of which most are relevant. Good level of knowledge and competences. | Limited HSA recruitment regarding education. Various HSA backgrounds, of which most are relevant. Varying knowledge and competences based on experience. | Broad HSA recruitment. Various HSA backgrounds, of which most are relevant. Varying knowledge and competences based on experience. | Broad HSA recruitment. Various HSA backgrounds, of which most are relevant. Varying knowledge and competences based on experience. |
|  |  | I | *Prom* | *n.a.* | *n.a.* | *n.a.* | *n.a.* | + | *n.a.* | *n.a.* | *n.a.* |
| Factors relating to the organization (PHSs) | | | | | | | | | | | |
| ***Overall score*** |  |  | *Score* | *-* | *-* | *-* | *-* | *+* | *+* | *+* | *++* |
|  |  | I | *Prom* | - | - | - | - | + | - (now, previously +) | + | + |
|  |  |  |  |  |  |  |  |  |  |  |  |
| **F6 Organization policy** | Firmness position of HPS in PHS policy | I, D | *Score* | - | - | -- | -- | + | + | ++ | ++ |
|  |  |  | *Descr* | In the past, there was less attention for HPS in PHS policy due to reorganization and budget cuts. Currently, a firmer position of HPS in policy is developing. | The efforts of HSAs are not reflected by the positon of HPS in policy. A new policy is being developed, in which HPS will have a stronger position. | The efforts of HSAs are not reflected by the position of HPS in policy. | Due to many budget cuts and reorganizations, there is less attention for HPS in policy. There is no PHS vision on HPS support. | Policy explicitly includes HPS support. Due to a recent reorganization, there is somewhat less attention for HPS support as a consequence of focus on internal processes. | Policy explicitly includes HPS support. Often changes in policy, which is perceived as hindering. | HPS support has a long-term firm position in policy. In a recent new vision, HPS support is explicitly included. | HPS support has a long-term firm position in policy. |
|  |  | I | *Prom* | - | *n.a.* | *n.a.* | - | - | - | + | *n.a.* |
| **F7 Management support** | Support from tactical and strategic levels | I | *Score* | + | + | - | - | + | ++ | + | ++ |
|  |  |  | *Descr* | Hindering influence due to staff turnover at tactical and strategic levels. Currently, there is support at tactical and strategic levels. | Hindering influence due to staff turnover at strategic level. Currently, there is strong support at tactical level and reasonable support at strategic level. | Strategic level is unaware of HPS. Tactical level offers support, but this is vulnerable due to staff turnover. | Hindering influence due to much staff turnover at strategic level. Currently, there is support at tactical level and limited support at strategic level. | Strong support at tactical level. Limited support at strategic level. | Strong support at tactical level. Strong support at strategic level, manager is actively involved. | Strong support at tactical level. Limited support at strategic level. | Strong support at tactical level. Strong support at strategic level, manager is actively involved. |
|  |  | I | *Prom* | - | *n.a.* | *n.a.* | - | - | *n.a.* | *n.a.* | *n.a.* |
| **F8 Internal support** | Support from other departments within the PHS | I | *Score* | + | -- | + | -- | - | - | ++ | + |
|  |  |  | *Descr* | There is support from other departments. HPS is starting to become well known in the PHS. | There is little support from other departments. With some departments, HPS is never discussed. | There is reasonable support from other departments, but colleagues don't yet easily make connections. | There is little support from other departments. The team mainly works independently. | There is reasonable support from other departments, but is can be improved. There is some competition among departments. | There is little support from other departments. HSAs are limitedly involved in policy changes. | There is support from all departments and from all individuals. There is good collaboration between HSAs and colleagues. | There is support from most departments, but it varies among departments. |
|  |  | I | *Prom* | - | - | - | - | *n.a.* | - | *n.a.* | *n.a.* |
|  |  |  |  |  |  |  |  |  |  |  |  |
| **F9 Capacity (numer of FTE)** | Number of fte per 100 schools (very little 0.00-0.12; little 0.13-0.50; much 0.50-1.00; very much 1.00-3.00), meeting school demand, capacity for extra tasks (e.g., recruitment) | I, E | *Score* | -- | -- | - | - | + | + | - | ++ |
|  |  |  | *Descr* | 0.11 fte of HSAs. School demand can't be met and there is no capacity for extra tasks. | 0.12 fte of HSAs. School demand can't be met and there is no capacity for extra tasks. | 0.96 fte of HSAs. School demand can't be met and there is no capacity for extra tasks. | 0.18 fte of HSAs. School demand can't be met and there is no capacity for extra tasks. | 2.26 fte of HSAs and this will increase. School demand can't be met. | 2.83 fte of HSAs. School demand can't be met. | 0.60 fte of HSAs. School demand can't be met and there is no capacity for extra tasks. | 2.16 fte of HSAs. School demand can be met, but there is no capacity for extra tasks. |
|  |  | I | *Prom* | - | - | *n.a.* | - | + | - | + | + |
| **F10 (Structural) budget** | Amount, fragmentation, structural | I, D | *Score* | -- | -- | - | + | ++ | ++ | ++ | ++ |
|  |  |  | *Descr* | Budget is very limited and almost exclusively exists of national grants. A small part is structural and several local governments provide additional funding. | Budget is very limited and almost exclusively exists of national grants. A small part is structural and several local governments provide additional funding. | Budget is limited and almost exclusively exists of temporary sources. It includes grants and additional funding by several local governments. | Budget is limited, but almost exclusively exists of structural sources from local governments. There is no dependence on national grants. | Considerable budget, which partly exists of structural sources from local governments. Also dependence on national grants and other funding. Next year, structural budget will increase. | Considerable budget, which exists of structural sources from most local governments. There is very limited dependence on national grants or other funding. | Considerable budget, which exists of structural sources from most local governments. There is very limited dependence on national grants or other funding. | Considerable budget, which exists of structural sources from most local governments. There is very limited dependence on national grants or other funding. |
|  |  | I | *Prom* | *n.a.* | *n.a.* | - | - | + | - | *n.a.* | *n.a.* |
| **F11 Learning organization** | Clear onboarding plan, following trainings, recurrent internal intervision, recurrent external intervision | I | *Score* | - | + | ++ | + | + | ++ | + | ++ |
|  |  |  | *Descr* | No onboarding plan. HSAs follow limited training. Limited internal intervision. Recurrent external intervision with another PHS. | No onboarding plan. Recurrent internal intervision. Time invested in further developing the way of working. | Clear and elaborate onboarding plan. HSAs regularly follow trainings. Recurrent intervision. Time invested in further developing the way of working. | Onboarding plan available. HSAs regularly follow trainings. Recurrent intervision. | Clear and elaborate onboarding plan. HSAs regularly follow trainings. Recurrent intervision. | Clear and elaborate onboarding plan. HSAs regularly follow trainings. Recurrent internal trainings. Recurrent intervision. Time invested in further developing the way of working. | Clear and elaborate onboarding plan. HSAs regularly follow trainings. Recurrent intervision, but mainly 1-on-1. | Clear and elaborate onboarding plan. Recurrent internal trainings. Recurrent intervision. Time invested in further developing the way of working. |
|  |  | I | *Prom* | *n.a.* | *n.a.* | *n.a.* | *n.a.* | *n.a.* | *n.a.* | *n.a.* | + |
| Factors relating to collaboration | | | | | | | | | | | |
| ***Overall score*** |  |  | *Score* | *-* | *-* | *-* | *+* | *+* | *+* | *++* | *++* |
|  |  | I | *Prom* | - | - | - | + | +/- | +/- | +/- | + |
|  |  |  |  |  |  |  |  |  |  |  |  |
| **F12 Shared vision** | Shared vision with youth health care department, local governments, and other stakeholders | I, D | *Score* | + | - | -- | + | + | - | ++ | ++ |
|  |  |  | *Descr* | Shared vision with youth health care. Difference in vision with local governments, although this is improving. Shared vision with other stakeholders. | Mostly shared vision with youth health care. Difference in vision with local governments. Stakeholders generally have a more narrow vision. | Difference in vision with youth health care, but this is improving. Varying vision across local governments, but often different. Stakeholders generally have a more narrow vision. | Difference in vision with youth health care. Varying visions of local governments and stakeholders, but it is generally shared. | Mostly shared vision with youth health care. Varying vision across local governments, but often shared. Stakeholders have a reasonably shared vision, although more narrow. | Difference in vision with youth health care. Varying visions of local governments and stakeholders. Some have a shared vision and some to a limited extent. | Shared vision with youth health care, although collaboration is limited. Strong shared vision with local governments and stakeholders, but requires continued attention. | Mostly shared vision with youth health care. Strong shared vision with local governments and stakeholders, but requires continued attention. |
|  |  | I | *Prom* | *n.a.* | + | + | - | *n.a.* | *n.a.* | - | *n.a.* |
| **F13 Coordination** | Coordination with other PHS-departments, coordination at tactical and strategic levels between PHS, local governments, and education, coordination with other stakeholders | I | *Score* | - | *--* | *--* | - | + | + | + | ++ |
|  |  |  | *Descr* | Good coordination within PHS. Limited coordination at tactical and strategic levels with local governments. Limited coordination with education. Limited coordination with other stakeholders. | Varying coordination within PHS. Limited coordination at tactical and strategic levels with local governments. Limited coordination with education. Limited coordination with other stakeholders. | Limited coordination within PHS, but this is developing. Limited coordination at tactical and strategic levels with local governments. Limited coordination with education. Limited coordination with other stakeholders. | Limited coordination within PHS. Limited coordination at tactical and strategic levels with local governments. Limited coordination with education. Varying coordination with other stakeholders. | Coordination within PHS. Coordination at tactical and strategic levels with local governments. Limited coordination with education. Varying coordination with other stakeholders. | Limited coordination within PHS, but developing. Coordination at tactical and strategic levels with local governments. Limited coordination with education. Varying coordination with other stakeholders. | Limited coordination within PHS. There is coordination at tactical and strategic levels with local governments. Limited coordination with education. Varying coordination with other stakeholders. | Coordination within PHS. There is coordination at tactical and strategic levels with local governments. Coordination with education was attempted in the past, but currently limited. Good coordination with other stakeholders. |
|  |  | I | *Prom* | *n.a.* | - | *n.a.* | *n.a.* | *n.a.* | *n.a.* | *n.a.* | *n.a.* |
| **F14 Division of responsibilities** | Clarity of division of responsibilities, both internally and externally | I | *Score* | - | - | -- | - | + | + | + | + |
|  |  |  | *Descr* | Internal division is clear. With stakeholders, it varies and there is a desire for clarification. | Internal division not always clear. With stakeholders, it varies and there is a desire for clarification. | Internal division not always clear. With stakeholders, it is often unclear and there is a desire for clarification. | Internal division is clear. With stakeholders, it varies and there is a desire for clarification. | Internal division is clear. With stakeholders, it varies but is mostly clear. | Internal division is mostly clear. With stakeholders, it is mostly clear. | Internal division reasonably clear. With stakeholders, it varies but is mostly clear. | Internal division is clear. With stakeholders, it varies but is mostly clear. |
|  |  | I | *Prom* | - | *n.a.* | *n.a.* | - | - | *n.a.* | *n.a.* | - |
|  |  |  |  |  |  |  |  |  |  |  |  |
| **F15 Communication structure** | Clear communication structure with stakeholders, easy contact with stakeholders | I | *Score* | -- | - | + | ++ | ++ | + | ++ | ++ |
|  |  |  | *Descr* | No regular communication structure. Easy contact with some stakeholders in some municipalities. | Varying communication structures, but mostly not in place. Easy contact with some stakeholders in some municipalities. | Varying communication structures, but for about half of the stakeholders in place. Easy contact with stakeholders. | Varying communication structures, but for most stakeholders in place. Easy contact with stakeholders. | Varying communication structures, but for most stakeholders in place. Easy contact with stakeholders. | Limited communication structures, varying across municipalities. Easy contact with stakeholders. | Varying communication structures, but for most stakeholders in place. Easy contact with stakeholders. | Varying communication structures, but for most stakeholders in place. Easy contact with stakeholders. |
|  |  | I | *Prom* | *n.a.* | - | *n.a.* | *n.a.* | + | *n.a.* | - | *n.a.* |
| **F16 Formalization** | Agreements with stakeholders are formalized | I, D | *Score* | + |  |  |  | ++ |  |  | ++ |
|  |  |  | *Descr* | Agreements with some stakeholders are formalized. | *n.a.* | *n.a.* | *n.a.* | Agreements with most stakeholders are formalized. | *n.a.* | *n.a.* | Agreements with most stakeholders are formalized. |
|  |  | I | *Prom* | *n.a.* | *n.a.* | - | - | - | *n.a.* | *n.a.* | *n.a.* |
| **F17 Monitoring** | Recurrent formal evaluation, recurrent informal evaluation |  | *Score* | -- |  | + | + | + |  |  | + |
|  |  | I | *Descr* | There is almost no monitoring. | *n.a.* | There is almost no formal evaluation, but problems are often discussed. | There is limited formal evaluation, but problems are often discussed. | There is almost no formal evaluation, but problems are often discussed. | *n.a.* | *n.a.* | It varies across stakeholders, but formally conducted for most. |
|  |  | I | *Prom* | *n.a.* | *n.a.* | *n.a.* | *n.a.* | *n.a.* | *n.a.* | *n.a.* | *n.a.* |
| **F18 Partner quality** | Number of prominent partners locally, regionally, and nationally (few 0-5; reasonably many 6-8; many 9-11) and variation across topics |  | *Score* | - | - | ++ | ++ | + | -- | ++ | ++ |
|  |  | I | *Descr* | Few local, many regional, and reasonably many national partners. Good variation across topics. | Few local, reasonably many regional, and few national partners. Good variation across topics. | Many local, many regional, and few national partners. Good variation across topics. | Reasonably many local, regional, and national partners. Good variation across topics. | Reasonably many local and regional, and few national partners. Good variation across topics. | Few local and regional, and reasonably many national partners. Limited variation across topics. | Many local and few regional and national partners. Good variation across topics. | Many local, reasonably many regional, and many national partners. Good variation across topics. |
|  |  | I | *Prom* | *n.a.* | *n.a.* | *n.a.* | *n.a.* | *n.a.* | *n.a.* | *n.a.* | *n.a.* |
| **F19 History with partners** | Length of history, quality of history |  | *Score* | ++ |  |  | ++ |  | + |  | + |
|  |  | I | *Descr* | Long and good history with many partners. | *n.a.* | *n.a.* | Long and good history with many partners. | *n.a.* | Long and good history with most partners. | *n.a.* | Long and good history with most partners. |
|  |  | I | *Prom* | *n.a.* | *n.a.* | *n.a.* | + | *n.a.* | *n.a.* | *n.a.* | *n.a.* |
| Factors relating to wider context | | | | | | | | | | | |
| ***Overall score*** |  |  | *Score* | *+* | *+* | *+* | *+* | *-* | *+* | *-* | *+* |
|  |  | I | *Prom* | +/- | + | - | +/- | + | *n.a.* | + | + |
|  |  |  |  |  |  |  |  |  |  |  |  |
| **F20 Healthy School Program** | Public awareness, structure, and materials of the program |  | *Score* |  | + | ++ | + | + | + | - | ++ |
|  |  | I | *Descr* | *n.a.* | The continued awareness is stimulating, although the added value can be communicated about more clearly. | The structure of the program, the grants, and the awareness are stimulating. | The materials and awareness of the program are stimulating, although it could be positioned stronger at a national level. | The grants, flexibility, and awareness are stimulating. The demand-driven structure is difficult in convincing local governments. | The grants are stimulating, but the complexity of the grant application process is hindering. | The non-commital nature causes HSAs to not being able to mandate schools, which leads to dependence on individuals within a school. | The structure, grants, and awareness are stimulating. |
|  |  | I | *Prom* | *n.a.* | *n.a.* | *n.a.* | + | + | *n.a.* | *n.a.* | + |
| **F21 Size of work area** | Number of municipalities (small <12; medium 12-15; large >15), number of inhabitants (small (<550.000; medium 550.000-750.000; large >750.000), number of schools (small <300; medium 300-400; large >400) |  | *Score* | + | ++ | ++ | ++ | -- | + | - | + |
|  |  | E | *Descr* | Medium number of municipalities, inhabitants, and schools | Small number of municipalities, inhabitants, and schools | Small number of municipalities, inhabitants, and schools | Medium number of municipalities, small number of inhabitants and schools | Large number of municipalities, inhabitants, and schools. | Small number of municipalities, large number of inhabitants and schools. | Large number of municipalities, medium number of inhabitants, small number of schools. | Medium number of municipalities, inhabitants, and schools. |
|  |  | I | *Prom* | *n.a.* | *n.a.* | *n.a.* | *n.a.* | *n.a.* | *n.a.* | *n.a.* | *n.a.* |
| **F22 Attention for health promotion** | Attention at local and national levels |  | *Score* | + | + | + | + | + | ++ | + | ++ |
|  |  | I | *Descr* | Reasonably much attention locally. Stimulating influence of national attention. | Stimulating influence of national attention. | Stimulating influence of national attention. | Stimulating influence of national attention. | Stimulating influence of national attention. | Much attention locally. Stimulating influence of national attention. | Stimulating influence of national attention, although this can be positioned stronger. | Much attention locally. Stimulating influence of national attention. |
|  |  | I | *Prom* | *n.a.* | *n.a.* | *n.a.* | *n.a.* | *n.a.* | *n.a.* | + | *n.a.* |
|  |  |  |  |  |  |  |  |  |  |  |  |
| **F23 Covid-19** | Influence of covid-19 on HPS support |  | *Score* | - | - | - | - | -- | -- | - | -- |
|  |  | I | *Descr* | Communication with partners was more difficult. There was also stimulating influence due to more attention for health promotion. | HSAs were less in touch with schools. There was also stimulating influence due to more attention for health promotion. | HSAs were less in touch with schools, mainly due to less personnel. There was also stimulating influence due to more attention for health promotion. | HSAs were less in touch with schools. There was also stimulating influence due to more attention for health promotion. | Internal communication was difficult. HSAs were less in touch with schools. There was more attention for health promotion, but no clear stimulating influence. | Internal communication was difficult. HSAs were less in touch with schools. | HSAs were less in touch with schools. There was also stimulating influence due to more attention for health promotion. | Internal communication was difficult. HSAs were less in touch with schools. |
|  |  | I | *Prom* | *n.a.* | *n.a.* | *n.a.* | *n.a.* | *n.a.* | *n.a.* | *n.a.* | *n.a.* |
| **F24 Other region-specific factors** | Other region-specific factors that influenced HPS support |  | *Score* | - | - | - |  | - | + | + | - |
|  |  | I | *Descr* | Municipal policy on HPS can be improved. | Youth health care physicians spend less time at schools, which hinders their ambassador role for HPS. | Municipal budget sources vary between PHS and stakeholders: either from education or from health. Municipal policy varies. | *n.a.* | Other health promotion programs can hinder HPS, as those advisers also support schools outside of their expertise. | Municipal policy is stimulating. | Because of an existing project, there are many existing communication structures. | Municipal policy is stimulating, but could be stronger. |
|  |  | I | *Prom* | *n.a.* | *n.a.* | *n.a.* | *n.a.* | *n.a.* | *n.a.* | *n.a.* | *n.a.* |

*Note: I = interviews, D = documents, E = existing data, Descr = description of data, Prom = prominent factor according to participants, HSA = Healthy School Adviser, PHS = Public Health Service, HPS = Health Promoting School, FTE = full-time equivalent, n.a. = the factor or its perceived prominence was not addressed by participants.*
